# Supplementary material for: A 5-hydroxymethylcytosine DNA glycosylase provides defense against T-even bacteriophages
Source: bioRxiv. 2026 Feb 25:2026.02.25.707755. Preprint. [Version 1] doi: 10.64898/2026.02.25.707755 (PMC13160076; doi:10.64898/2026.02.25.707755)
Supplement: Supplement 1 [file NIHPP2026.02.25.707755v1-supplement-1.pdf]

bioRxiv preprint doi: https://doi.org/10.64898/2026.02.25.707755; this version posted February 25, 2026. The copyright holder for this preprint (which was not certified by peer review) is the author/funder, who has granted bioRxiv a license to display the preprint in perpetuity. It is made available under aCC-BY 4.0 International license.

SUPPLEMENTARY FIGURES

Figure S1. Isolation of Brig3 from an eDNA library. (A) Ten-fold serial dilutions of T5 or T4(5hmC) phage on lawns of *E. coli* EC100 cells generated from colonies sampled randomly from the eDNA library population that survived T4(5hmC) infection. Cells harboring an empty pWEB cosmid were used as a negative control. A representative result for a surviving colony that did not contain a *bona fide* immunity gene in the eDNA is shown. Plaquing on a lawn of cells that originated from a surviving colony that harbored a cosmid carrying immunity genes is also shown. **(B)** Genes present within the 35.4 kb soil metagenomic DNA fragment that provided T4 immunity. Subcloned regions (fragments F1-F6) are indicated. Putative defense genes are shown in grey. Genes labeled "*1*" and "*2*" correspond to *ORF1* (Brig3) and *ORF2* (BapA), respectively. **(C)** Ten-fold serial dilutions of phage T4 on lawns of *E. coli* EC100 carrying pWEB or cosmids containing fragments F1-F6. **(D)** Amino acid alignment of Brig1 and Brig3. (*), (:) and (.) mark identical, strongly conserved and weakly conserved residues, respectively.

Figure S2. Infection of cultures expressing Brig3 and/or BapA. (A-D) Growth of *E. coli* EC100 that harbor the pWEB cosmid carrying wild-type and stop codon mutant versions of *ORF1* and/or *ORF2*, measured by optical density at 600 nm (OD_{600}) either **(A)** uninfected, or after infection with **(B)** T4(5hmC) at MOI 10, **(C)** T4(wt) at MOI 0.1 or **(D)** T4(wt) at MOI 1.0. The mean ± S.D. for 3 biological replicates is shown. **(E-H)** Growth of *E. coli* EC100 that harbor the pWEB cosmid as a negative control or plasmids over-expressing either *ORF1* or *ORF1/ORF2*, measured by optical density at 600 nm (OD_{600}) after infection with **(E)** T4(5hmC) at MOI 1.0, **(F)** T4(5hmC) at MOI 10, **(G)** T4(wt) at MOI 0.1 or **(H)** T4(wt) at MOI 1.0. The mean ± S.D. for 3 biological replicates is shown. **(I)** Quantitative PCR analysis of T4(5hmC) DNA through amplification of the *gp42* gene. Viral DNA was extracted from infected *E. coli* EC100 cells carrying pWEB or p*ORF1* at 2, 4 and 8 minutes after the addition of phage at an MOI of 1. Fold-change values were calculated relative to the pWEB 2-minute time point. Mean ± SD values are reported for three independent experiments; *p*-values are reported for multiple unpaired Student T-tests.

Figure S3. Brig3 immunity against diverse T-even phages. (A) Growth of *E. coli* EC100 that harbor the pWEB cosmid carrying wild-type and stop codon mutant versions of Brig3 and/or BapA, measured by optical density at 600 nm (OD_{600}) after infection with T2 at MOI 10. **(B)** Same as **(A)** but after T6 infection. **(C)** Ten-fold serial dilutions of T4 mutants lacking either α- or β-glucosyl transferase, Δ*a-gt* or Δ*b-gt*, respectively, on lawns of *E. coli* EC100 that harbor the pWEB cosmid encoding Brig3 or Brig3-BapA, or a plasmid that over-expresses (OE) these proteins.

Figure S4. Brig3 introduces abasic sites in ssDNA oligonucleotides containing 5hmC. (A) Size-exclusion chromatography (SEC) analysis of Brig3-His_6 purified through nickel affinity, using a Superdex 75 Increase column. **(B)** SDS-PAGE of the fraction corresponding to the SEC peak shown in **(A)**, visualized using Coomassie stain. **(C)** PAGE of 5hmC-containing oligonucleotides incubated with ten-fold concentrations of Brig3 at 37°C for 30 minutes, with and without heating in the presence of NaOH for 30 minutes. Gels were stained with ethidium bromide. L, ssDNA size ladder. **(D)** Same as **(C)** but including a reaction in which Brig3 treatment was followed by incubation with 50

bioRxiv preprint doi: https://doi.org/10.64898/2026.02.25.707755; this version posted February 25, 2026. The copyright holder for this preprint (which was not certified by peer review) is the author/funder, who has granted bioRxiv a license to display the preprint in perpetuity. It is made available under aCC-BY 4.0 International license.

units of Endonuclease IV at 37°C for 4 hours. **(E)** Calculated average masses, in daltons (Da), of the different 18-nucleotide oligonucleotides used for mass spectrometry. The central position (red “X”) is occupied by either cytosine (C), 5-hydroxymethylcytosine (5hmC) or α- or β-glucosyl-5hmC (α-glc-5hmC or β-glc-5hmC, respectively). **(F)** Deconvoluted zero-charge mass spectra from high resolution mass spectrometry of oligonucleotides harboring the nucleobases shown on the right at the central position, incubated with or without Brig3 at 37°C overnight. The masses of major peaks are indicated in red.

Figure S5. Crystal structure of wild-type apo-Brig3. (A) Crystal structure of wild-type Brig3, displaying a mixed α/β fold composed of nine α-helices and six β-strands. The curved, mixed β-sheet core and the α9-β6 insertion loop (in red) are labeled. **(B)** Crystal structure of alkyladenine DNA glycosylase (AAG), which also features a mixed α/β fold and shares a similar shape with Brig3 (PDB 1EWN). **(C)** Catalytic pocket of Brig3, lined with several aromatic residues. Key residues are labeled. **(D)** Active site of AAG, showing the flipped base ethenoadenine (εA) situated in the aromatic-lined pocket. Key residues are labeled.

Figure S6. Crystal structure of wild-type Brig3 with a dsDNA substrate. (A) Sequence of the 12-mer dsDNA used for co-crystallization with Brig3, the 5-hydroxymethylcytosine (5hmC) nucleobase is shown in red. **(B)** Charge distribution map of the Brig3:dsDNA complex. Residues R197 and R140 interact with opposite DNA backbones, positioning the dsDNA substrate in a basic region of Brig3. N225 inserts into the abasic site and makes hydrogen bonds with the guanine that originally paired with 5hmC, an interaction that stabilizes the product of the base excision reaction. **(C)** Stable hydrogen bonds detected between residues R140, A174, E175, R197, G229, T230, and N233 with the DNA phosphate backbone and/or nucleobases surrounding the abasic site (AP). **(D)** Stable hydrogen bonds formed between residue N225, which is inserted into the abasic site (apurinic/apyrimidinic site, AP), and the adjacent (G_7) and opposite (G_{19}) nucleobases. **(E)** Schematic of specific interactions between Brig3 and the 12-mer dsDNA substrate shown in **(A)**. Solid and dotted lines indicate interactions via the amino acid side chain or the peptide backbone, respectively. **(F)** Structural alignment between the apo (silver) and dsDNA-bound (gold) forms of Brig3, with an RMSD of 0.361 Å for the protein components. The only notable difference shown in the boxed panel is found in the α9-β6 insertion loop, which is marked in red. **(G)** Region highlighted by the inset in **(F)** showing the most important difference in both structures: a shift in the α9-β6 insertion loop (displayed in red for the Brig3:dsDNA complex).

Figure S7. Crystal structure of $Brig3^{D127N}$ with a dsDNA substrate. (A) Wild-type Brig3 (gold) bound to a 12-mer dsDNA duplex (green) with the structural cavities highlighted in translucent grey. Residue D127 is shown and colored in orange. The inset shows a zoomed view of the biggest cavity (presumably the active site) and its proximity to D127. **(B)** Size-exclusion chromatography (SEC) analysis of $Brig3^{D127N}$-His_6 purified through nickel affinity, using a Superdex 75 Increase column. **(C)** SDS-PAGE of the fraction corresponding to the SEC peak shown in **(B)**, visualized using Coomassie stain. **(D)** Same as **(B)** but for purified $Brig3^{D127A}$-His_6. **(E)** Same as **(C)** but for the $Brig3^{D127A}$-His_6 peak shown in **(D)**. **(F)** PAGE of 5hmC-containing oligonucleotides either untreated or incubated with wild-type and mutant (D127N, D127A) versions of Brig3 at 37°C for 30

bioRxiv preprint doi: https://doi.org/10.64898/2026.02.25.707755; this version posted February 25, 2026. The copyright holder for this preprint (which was not certified by peer review) is the author/funder, who has granted bioRxiv a license to display the preprint in perpetuity. It is made available under aCC-BY 4.0 International license.

minutes, followed by heating in the presence of NaOH for 30 minutes. Gels were stained with ethidium bromide. L, ssDNA size ladder. **(G)** Sequence of the 12-mer dsDNA used for co-crystallization with Brig3^{D127N}, the 5-hydroxymethylcytosine (5hmC) nucleobase is shown in red. **(H)** Structural alignment of the catalytic pocket in wild-type (silver) and D127N (gold) Brig3, both in complex with a dsDNA substrate harboring an abasic site (apurinic/apyrimidinic site, AP) or a 5hmC nucleobase (in blue), respectively. **(I)** Structural alignment of wild-type (silver) and D127N (gold) Brig3, both in complex with a dsDNA substrate harboring an abasic site (AP, in silver) or a 5hmC nucleobase (in green), respectively, showing an RMSD of 0.396 Å. Protein structures exhibit minimal conformational changes, with the α9-β6 insertion loop showing the only noticeable shift. The changes in the dsDNA substrate include a shift of approximately 7 Å and 6 Å for the 5hmC-containing and complementary strands, as well as bending of 66° towards the Brig3^{D127N} protein. **(J)** Region highlighted by the inset in **(I)**, showing the change in the α9-β6 insertion loop of approximately 2 Å toward the phosphate backbone. **(K)** Stable hydrogen bonds formed between residue N225, which is inserted in place of the flipped 5hmC nucleobase, and the adjacent (G_7) and opposite (G_{19}) nucleobases. **(L)** Stable hydrogen bonds detected between residues R53, Y97, S139, A174, E175, R197, A224, G229, T230, and N233 with the DNA phosphate backbone and/or nucleobases surrounding the 5hmC nucleobase. **(M)** Schematic of specific interactions between Brig3^{D127N} and the 12-mer dsDNA substrate shown in **(G)**. Solid and dotted lines indicate interactions via the amino acid side chain or the peptide backbone, respectively.

Figure S8. Purification and activity of BapA. (A) Size-exclusion chromatography (SEC) analysis of BapA after purification through nickel affinity and cleavage of the His_6-SUMO tag, using a Superdex 75 Increase column. **(B)** SDS-PAGE of the fraction corresponding to the SEC peak shown in **(A)**, visualized using Coomassie stain. **(C)** In vitro assembly of BapA and Brig3-His_6 at a 1:1.5 molar ratio, incubated on ice for one hour, and applied to a Superdex 75 Increase 10/300 column. The two distinct peaks are labeled "*1*" and "*2*". **(D)** SDS-PAGE analysis of the SEC fractions encompassing both peaks. The separate elution profiles suggest that the two proteins do not form a stable complex under these in vitro assembly conditions. **(E)** Luminescence assay for glucose detection after incubation of genomic DNA extracted from different T4 phages with BapA overnight at 37ºC. RLU, relative luminescence units. Error bars represent the standard error of the mean; *p*-values are reported for a Ordinary One-Way ANOVA with multiple comparisons. **(F)** Ten-fold serial dilutions of different T4 phage stocks obtained after amplification in *E. coli* EC100(pBapA) hosts ("[pBapA]"), plated on lawns of *E. coli* EC100 that harbor the pWEB or pBrig3 cosmids. **(G)** Ten-fold serial dilutions of different T4 phage stocks obtained after amplification in *E. coli* EC100 hosts harboring either pBapA ("[pBapA]") or a control cosmid ([pWEB]), plated on lawns of *E. coli* EC100 that express Brig1.

Figure S9. BapA structural model and purification of mutants. (A) AlphaFold 3 structural model of BapA in complex with a dsDNA substrate that harbors a 5hmC nucleobase, colored according with pIDDT confidence. The flexible loop 186-196 shows the lowest prediction confidence, consistent with its expected structural flexibility. **(B)** Electrostatic surface representation of the predicted BapA:dsDNA complex, showing the DNA duplex accommodated within a positively charged channel. **(C)** Structural

bioRxiv preprint doi: https://doi.org/10.64898/2026.02.25.707755; this version posted February 25, 2026. The copyright holder for this preprint (which was not certified by peer review) is the author/funder, who has granted bioRxiv a license to display the preprint in perpetuity. It is made available under aCC-BY 4.0 International license.

comparison of BapA (orange) with an ASCH domain-containing protein from *Zymomonas mobilis*, *Zm*ASCH (green; PDB 5GUQ) showing a conserved β-barrel 1 topology (RMSD 1.108 Å). **(D)** Size-exclusion chromatography (SEC) analysis of $BapA^{D101N}$ after purification through nickel affinity and cleavage of the His_6-SUMO tag, using a Superdex 75 Increase column. **(E)** SDS-PAGE of the fraction corresponding to the SEC peak shown in **(D)**, visualized using Coomassie stain. **(F)** Same as **(D)** but for purified $BapA^{Q252A}$. **(G)** Same as **(E)** but for the $BapA^{Q252A}$ peak shown in **(F)**.

Figure S10. Structural comparison of Brig1:dsDNA and $Brig3^{D127N}$:dsDNA complexes. (A) Overlay of an AlphaFold 3-generated Brig1:dsDNA complex (silver; ipTM = 0.91, pTM = 0.93, the dsDNA contains a single 5hmC nucleobase) with the crystal structure of $Brig3^{D127N}$:dsDNA (gold). Structures are shown in two orientations. Both structures position the dsDNA duplex within a similar cleft, yielding an overall RMSD of 1.075 Å between the protein structures. The similarities in the geometry of DNA docking suggests a conserved mode of substrate recognition across members of the Brig family. The arrow indicates the shift in the DNA backbone caused by conformational changes in the three N-terminal α-helices of Brig1 and $Brig3^{D127N}$. **(B)** Detailed view of the catalytic pockets within the Brig1:dsDNA model (silver) and the $Brig3^{D127N}$:dsDNA (gold) crystal structure, highlighting residues involved in 5hmC recognition and catalysis. The 5hmC base is coordinated by Q81, W122, and Q147 in Brig1, and by E55, W100, and N127 in $Brig3^{D127N}$, which also employs a Mg^{2+} ion and a coordinated water molecule. Brig1 exhibits a larger, more open pocket that extends further from the DNA, suggesting a potentially expanded catalytic space for accommodating the α-glc-5hmC substrate.

Figure S11. **Characterization data of synthetic hm5dC-modified DNA**. Conditions: AE-HPLC Dionex DNApac column, 80 °C, 1 mL/min, 0-40% of buffer B within 30 min, Buffer A: Tris-HCl (25 mM) and NaClO4 (20 mM) in aq 20% ACN, pH 8.0; Buffer B: Tris-HCl (25 mM) and $NaClO_4$ (0.6 M) in aq 20% acetonitrile, pH 8.0. LC-ESI MS was performed on Finnigan LCQ Advantage Max in the negative-mode (C18 XBridge column (2.5 μm, 2.1 mm × 50 mm), flow rate of 0.1 mL/min, gradient 0 to 100% B at 30 °C (eluent A: 8.6 mM triethylamine, 100 mM 1,1,3,3,3-hexafluoroisopropanol in H_2O; eluent B: methanol).

bioRxiv preprint doi: https://doi.org/10.64898/2026.02.25.707755; this version posted February 25, 2026. The copyright holder for this preprint (which was not certified by peer review) is the author/funder, who has granted bioRxiv a license to display the preprint in perpetuity. It is made available under aCC-BY 4.0 International license.

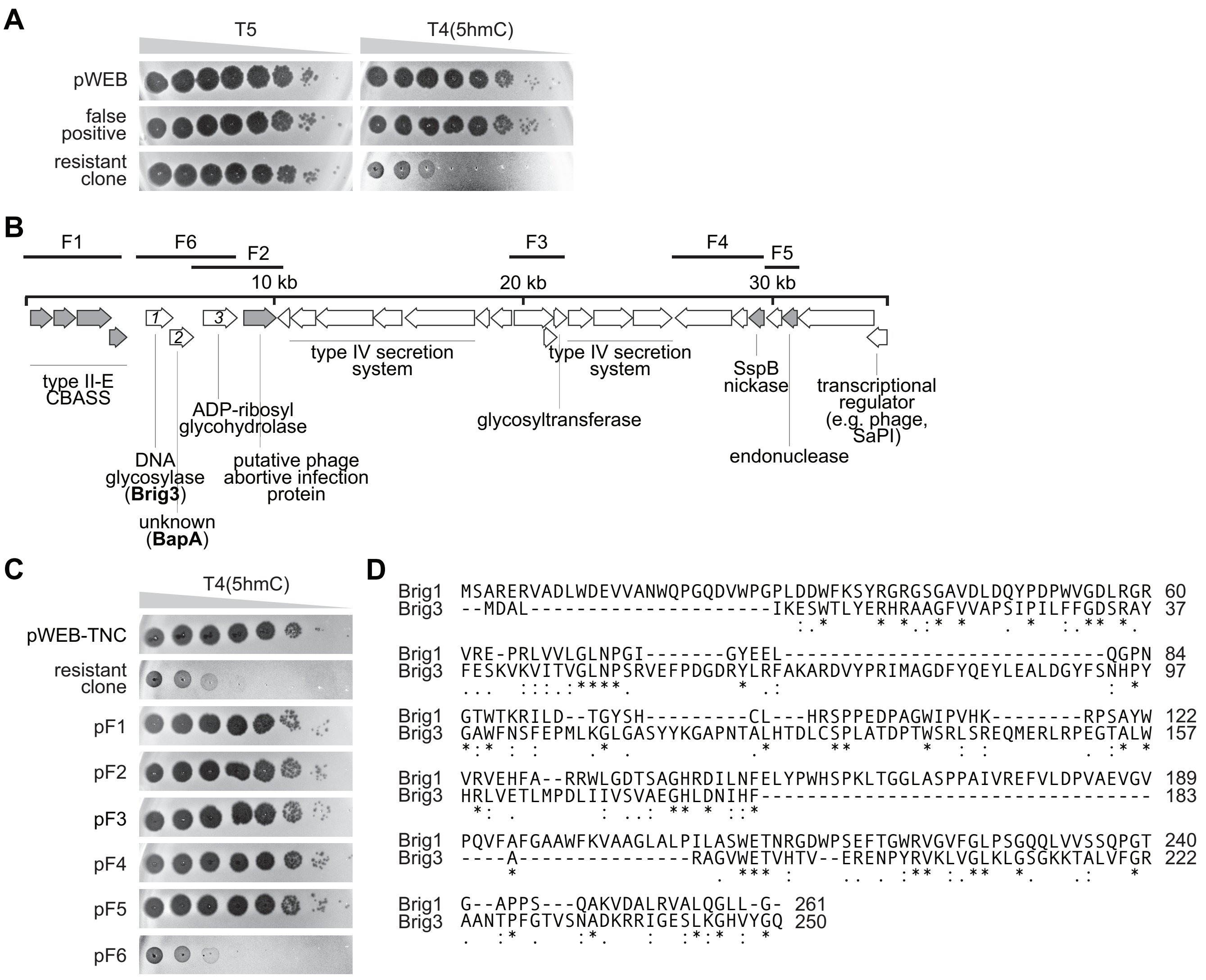

Fig. S1. Mejía-Pitta et al.

bioRxiv preprint doi: https://doi.org/10.64898/2026.02.25.707755; this version posted February 25, 2026. The copyright holder for this preprint (which was not certified by peer review) is the author/funder, who has granted bioRxiv a license to display the preprint in perpetuity. It is made available under aCC-BY 4.0 International license.

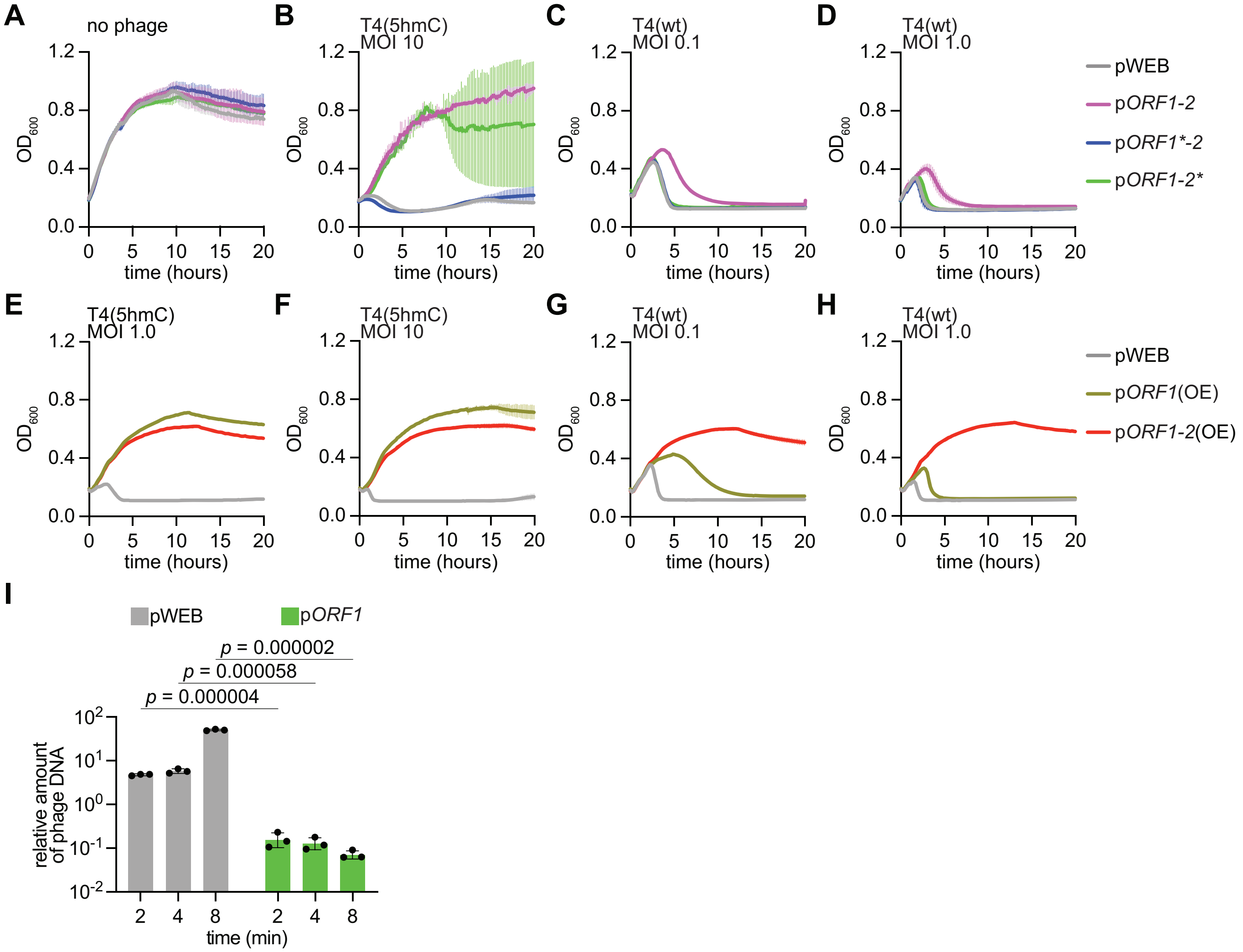

Fig. S2. Mejía-Pitta et al.

bioRxiv preprint doi: https://doi.org/10.64898/2026.02.25.707755; this version posted February 25, 2026. The copyright holder for this preprint (which was not certified by peer review) is the author/funder, who has granted bioRxiv a license to display the preprint in perpetuity. It is made available under aCC-BY 4.0 International license.

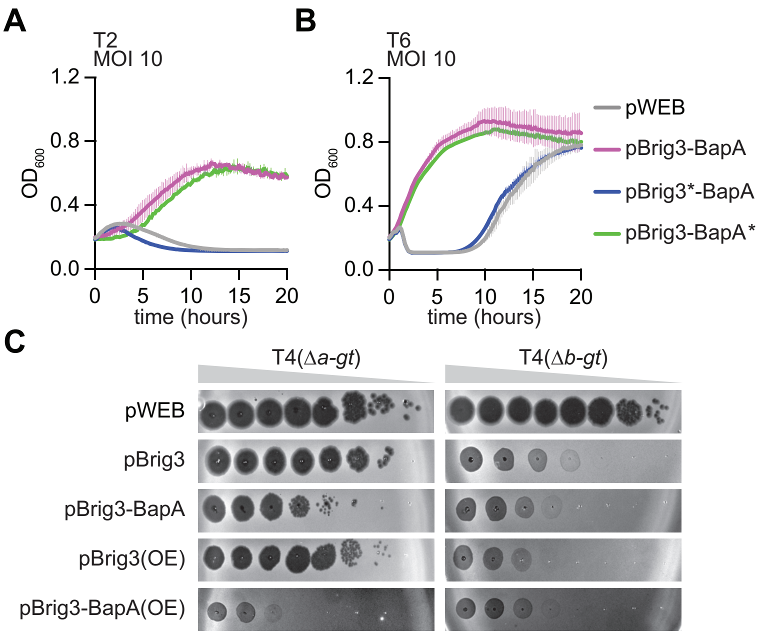

Fig. S3. Mejía-Pitta et al.

bioRxiv preprint doi: https://doi.org/10.64898/2026.02.25.707755; this version posted February 25, 2026. The copyright holder for this preprint (which was not certified by peer review) is the author/funder, who has granted bioRxiv a license to display the preprint in perpetuity. It is made available under aCC-BY 4.0 International license.

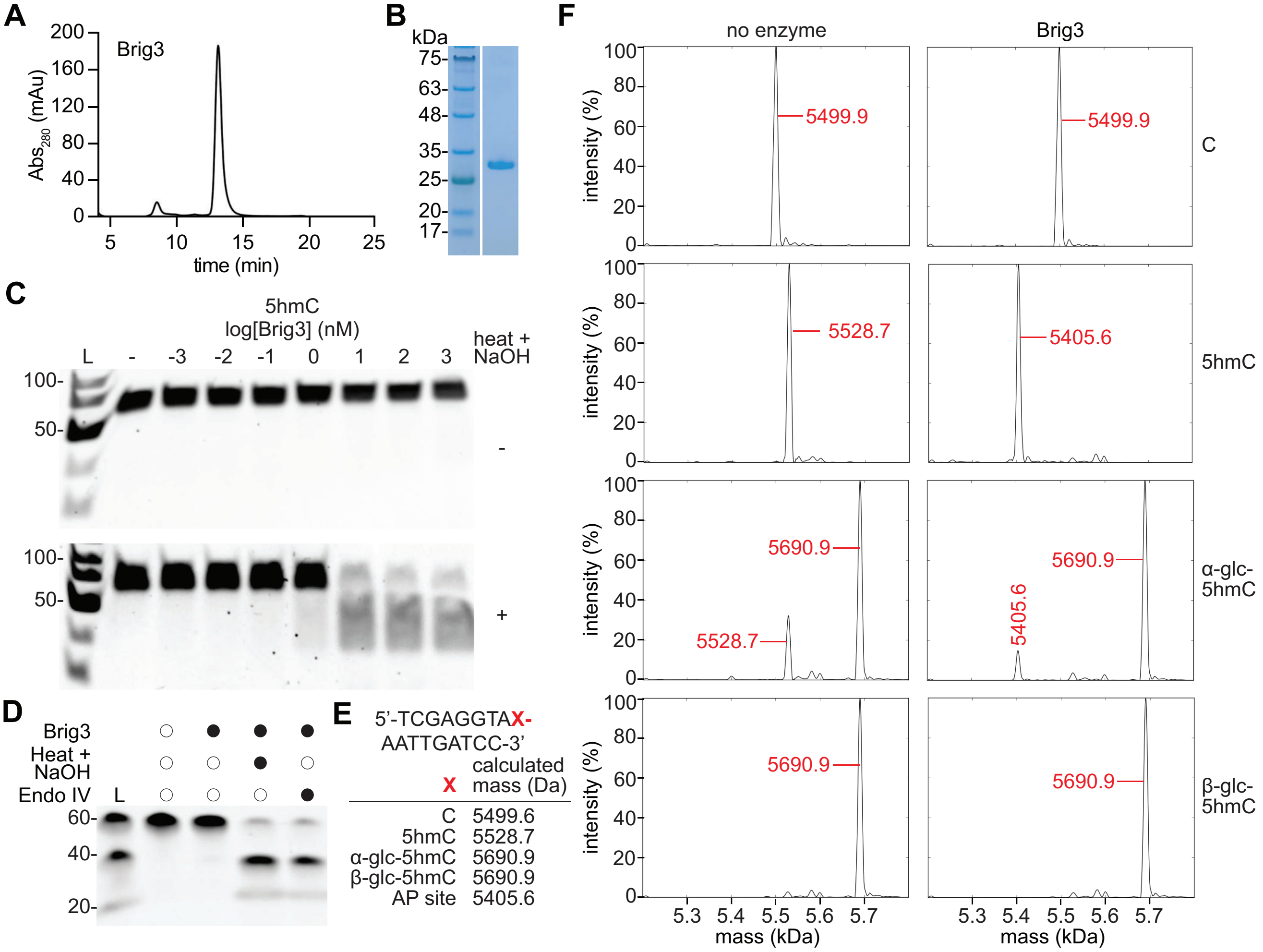

X	calculated mass (Da)
C	5499.6
5hmC	5528.7
α-glc-5hmC	5690.9
β-glc-5hmC	5690.9
AP site	5405.6

Fig. S4. Mejía-Pitta et al.

bioRxiv preprint doi: https://doi.org/10.64898/2026.02.25.707755; this version posted February 25, 2026. The copyright holder for this preprint (which was not certified by peer review) is the author/funder, who has granted bioRxiv a license to display the preprint in perpetuity. It is made available under aCC-BY 4.0 International license.

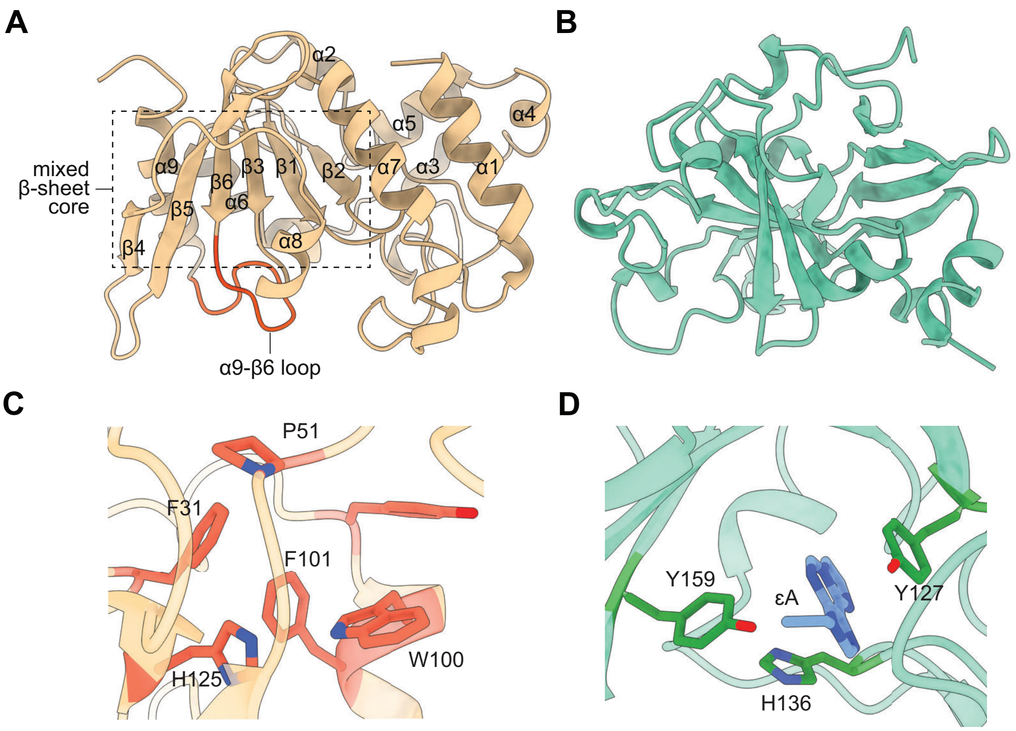

Fig. S5. Mejía-Pitta et al.

bioRxiv preprint doi: https://doi.org/10.64898/2026.02.25.707755; this version posted February 25, 2026. The copyright holder for this preprint (which was not certified by peer review) is the author/funder, who has granted bioRxiv a license to display the preprint in perpetuity. It is made available under aCC-BY 4.0 International license.

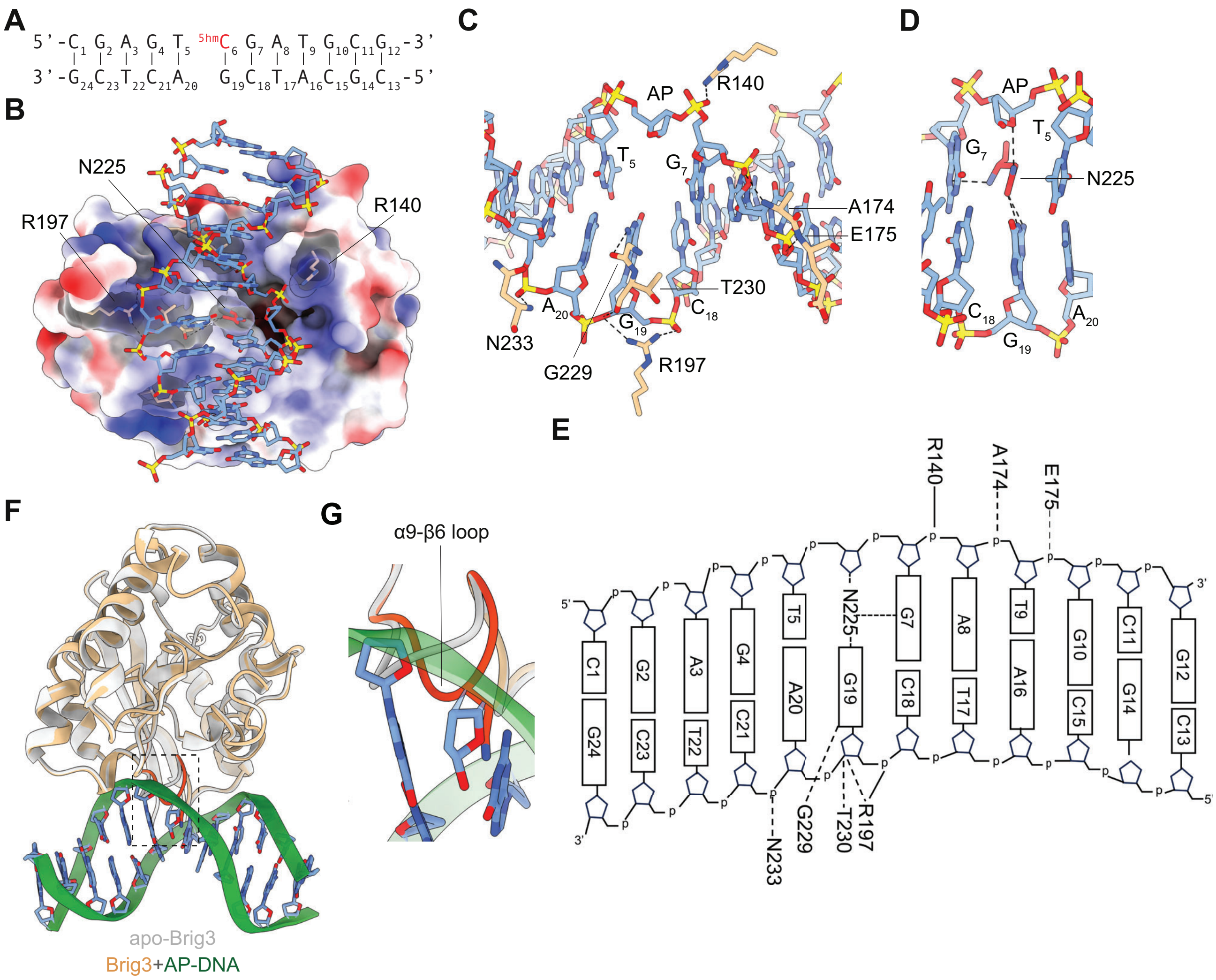

Fig. S6. Mejía-Pitta et al.

bioRxiv preprint doi: https://doi.org/10.64898/2026.02.25.707755; this version posted February 25, 2026. The copyright holder for this preprint (which was not certified by peer review) is the author/funder, who has granted bioRxiv a license to display the preprint in perpetuity. It is made available under aCC-BY 4.0 International license.

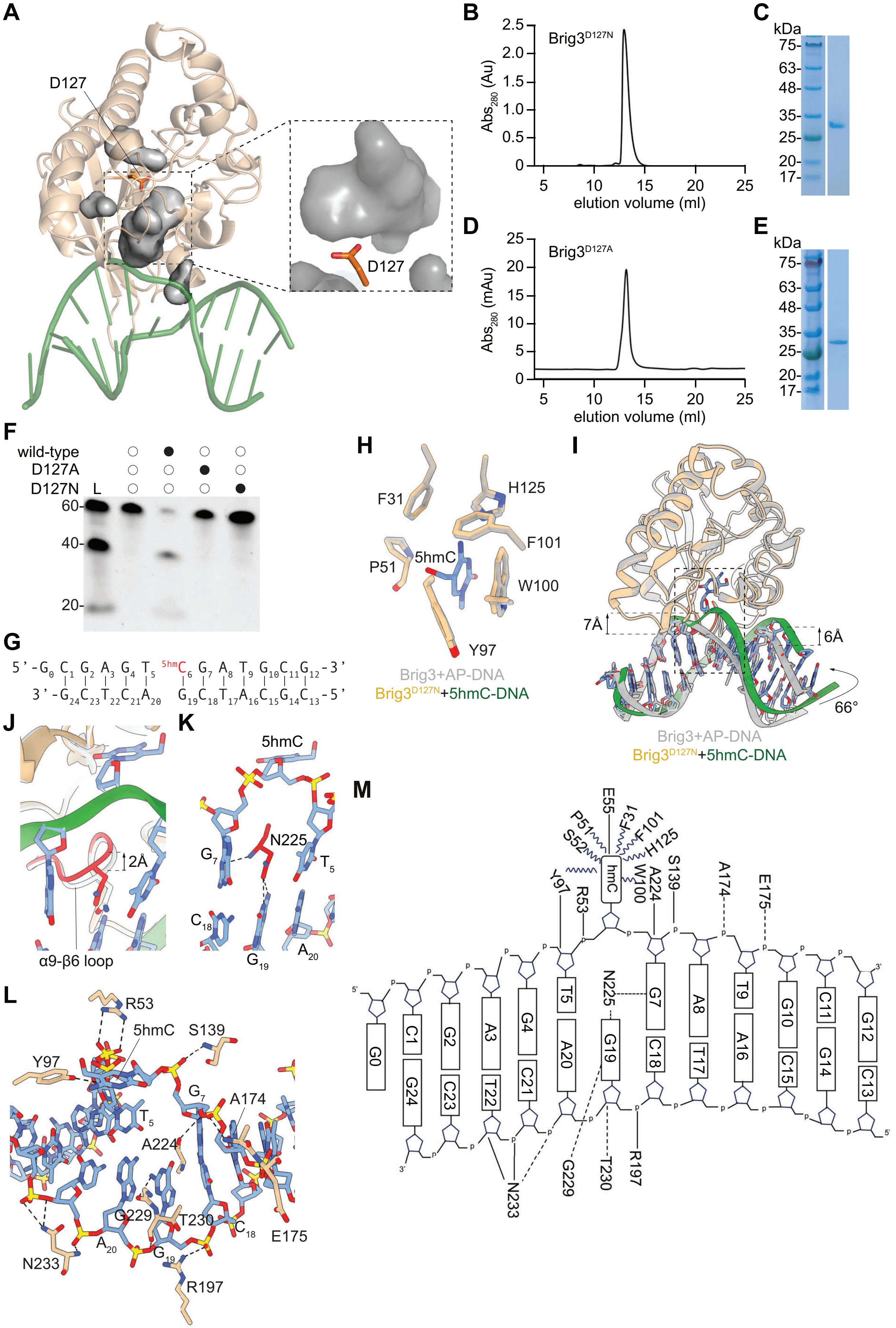

Fig. S7. Mejía-Pitta et al.

bioRxiv preprint doi: https://doi.org/10.64898/2026.02.25.707755; this version posted February 25, 2026. The copyright holder for this preprint (which was not certified by peer review) is the author/funder, who has granted bioRxiv a license to display the preprint in perpetuity. It is made available under aCC-BY 4.0 International license.

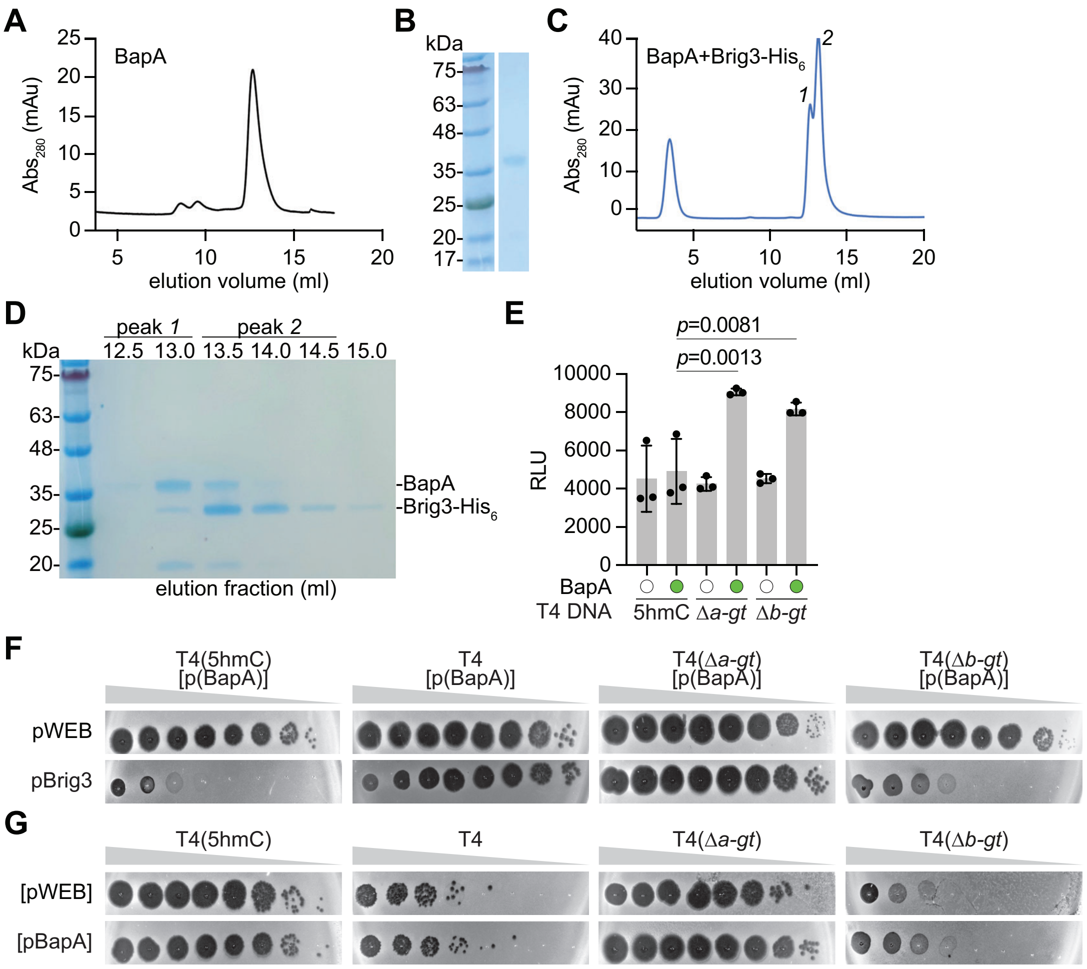

Fig. S8. Mejía-Pitta et al.

bioRxiv preprint doi: https://doi.org/10.64898/2026.02.25.707755; this version posted February 25, 2026. The copyright holder for this preprint (which was not certified by peer review) is the author/funder, who has granted bioRxiv a license to display the preprint in perpetuity. It is made available under aCC-BY 4.0 International license.

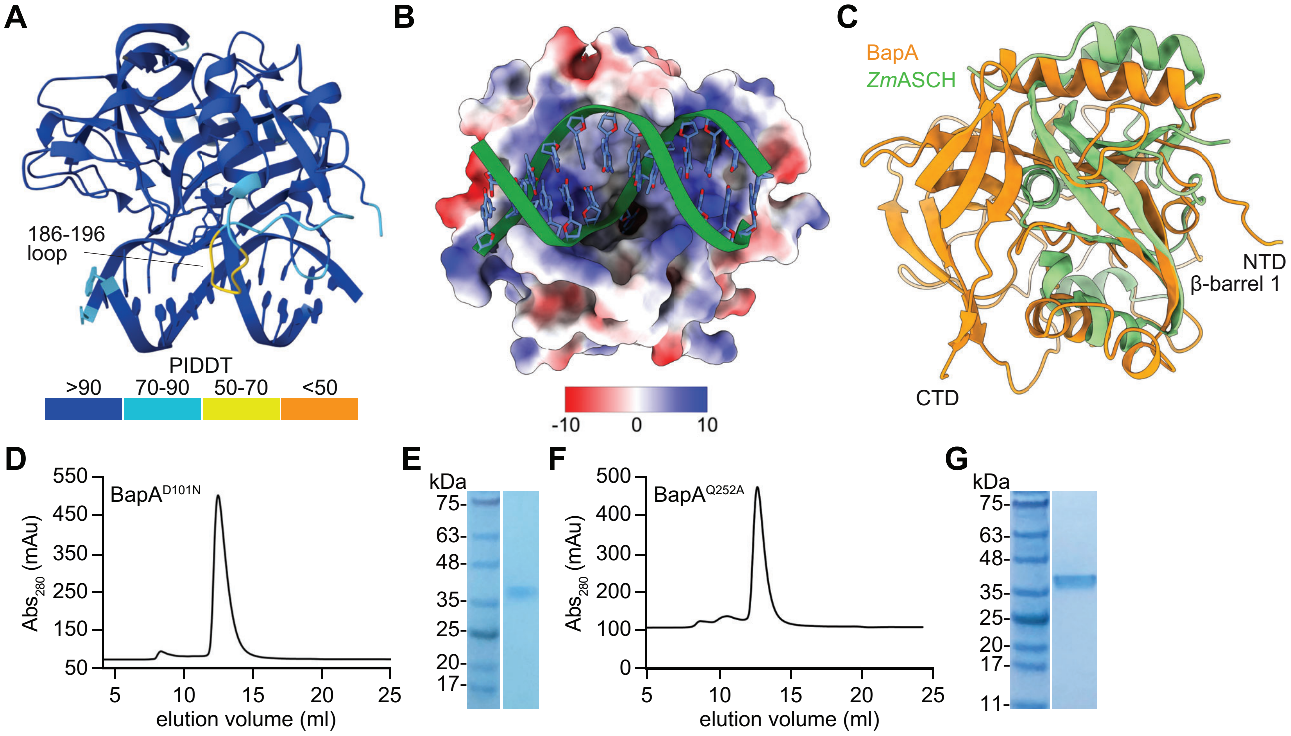

Fig. S9. Mejia-Pitta et al.

bioRxiv preprint doi: https://doi.org/10.64898/2026.02.25.707755; this version posted February 25, 2026. The copyright holder for this preprint (which was not certified by peer review) is the author/funder, who has granted bioRxiv a license to display the preprint in perpetuity. It is made available under aCC-BY 4.0 International license.

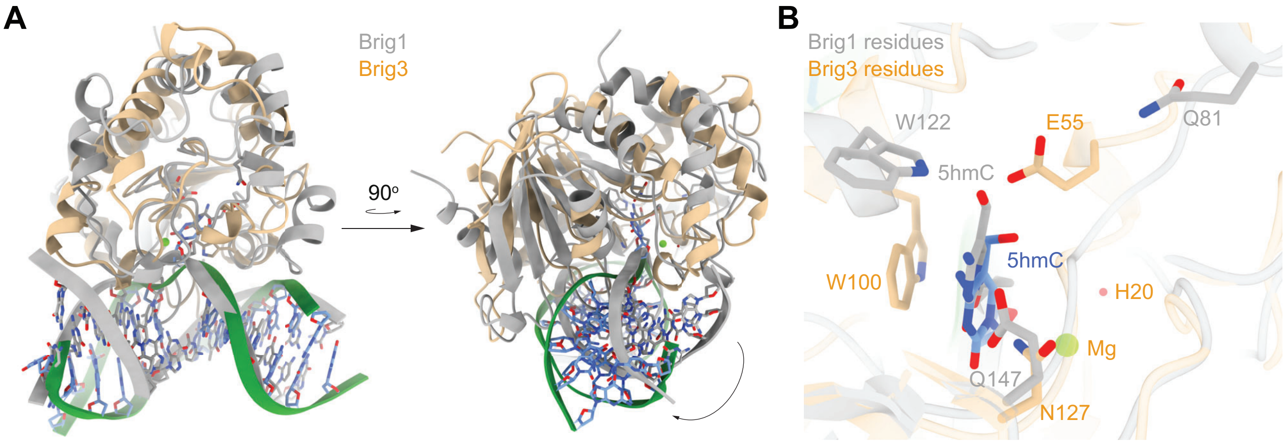

Fig. S10. Mejia-Pitta et al.

bioRxiv preprint doi: https://doi.org/10.64898/2026.02.25.707755; this version posted February 25, 2026. The copyright holder for this preprint (which was not certified by peer review) is the author/funder, who has granted bioRxiv a license to display the preprint in perpetuity. It is made available under aCC-BY 4.0 International license.

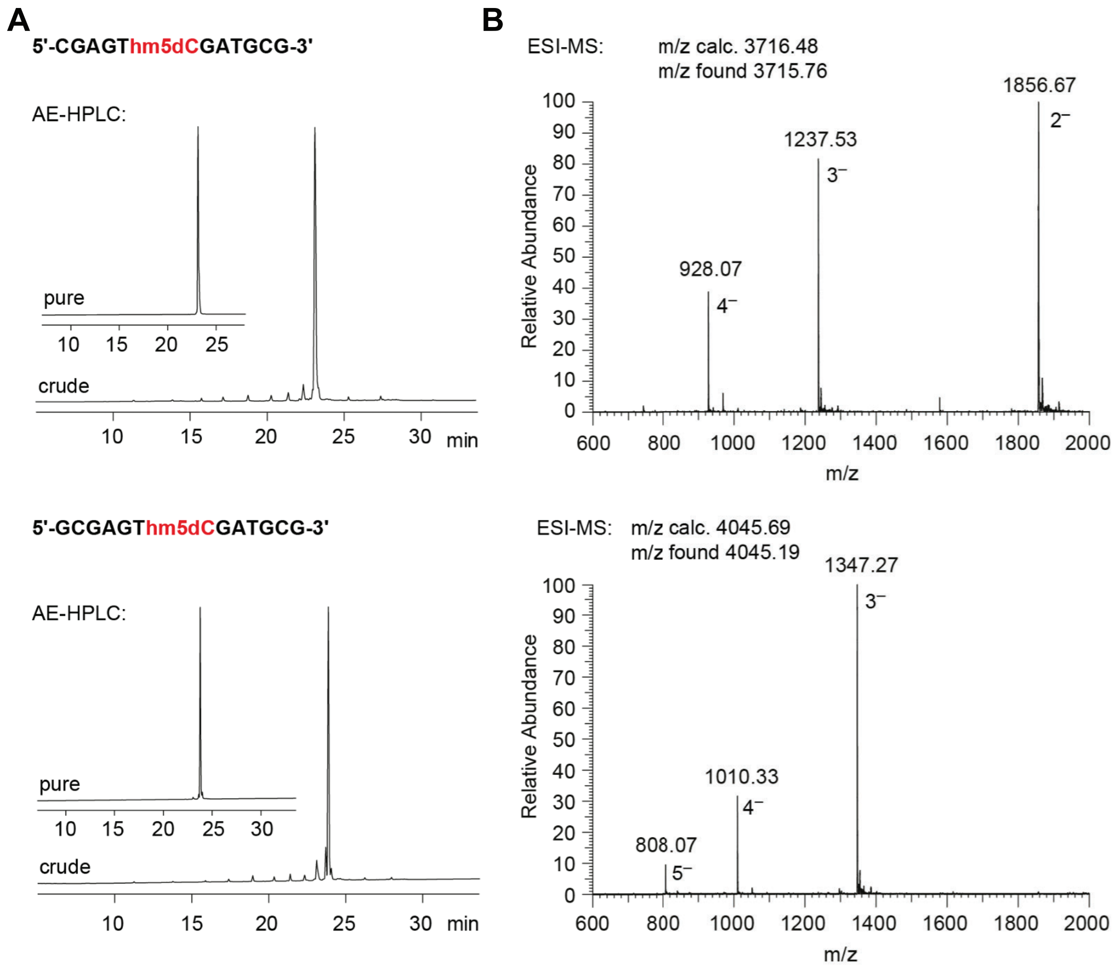

Fig. S11. Mejia-Pitta et al.

bioRxiv preprint doi: https://doi.org/10.64898/2026.02.25.707755; this version posted February 25, 2026. The copyright holder for this preprint (which was not certified by peer review) is the author/funder, who has granted bioRxiv a license to display the preprint in perpetuity. It is made available under aCC-BY 4.0 International license.

Table S1. Data collection and refinement statistics (molecular replacement)

	Brig3 apo	Brig3+12-mer dsDNA	Brig3 (D127N) +5'-overhang-containing 12-mer dsDNA
**Data collection**			
Space group	$P12_11$	$P2_12_12_1$	$C222_1$
Cell dimensions			
a, *b*, *c* (Å)	97.323, 95.339, 110.043	65.885, 73.479, 115.689	90.8, 96.839, 79.173
α, β, γ (°)	90, 100.909, 90	90, 90, 90	90, 90, 90
Resolution (Å)	47.67-2.894 (2.998-2.894)	45.45-1.76 (1.823-1.76)	33.12-1.61 (1.668-1.61)
R_{sym} or R_{merge}	9.3e-18 (1.249e-17)	0.02845 (0.8354)	0.06337 (1.112)
I / σI	7.85 (2.59)	11.51 (0.84)	5.02 (0.53)
Completeness (%)	97.05 (90.66)	99.68 (88.78)	99.33 (88.90)
Redundancy	1.0 (1.0)	2.0 (2.0)	2.0 (2.0)
**Refinement**			
Resolution (Å)	2.9	1.8	1.6
No. reflections	42963 (3950)	99986 (11097)	40417 (4451)
R_{work} / R_{free}	0.2063/0.2854	0.213/0.243	0.207/0.239
No. atoms	15909	4655	2694
Macromolecules	15909	4420	2508
Ligand/ion	0	12	1
Water	0	223	185
B-factors	39.47	37.44	36.49
Macromolecules	39.47	36.10	28.01
Ligand/ion	0	29.89	39.32
Water	0	38.09	35.41
R.m.s. deviations			
Bond lengths (Å)	0.01	0.008	0.013
Bond angles (°)	1.24	1.12	1.47

*Number of xtals for each structure: One crystal. *Values in parentheses are for highest-resolution shell.
